# PD-1 Inhibitors in the Advanced Esophageal Cancer

**DOI:** 10.3389/fphar.2019.01418

**Published:** 2019-11-29

**Authors:** Ye Hong, Zhen-Yu Ding

**Affiliations:** Department of Biotherapy, Cancer Center, West China Hospital, West China Medical School, Sichuan University, Chengdu, China

**Keywords:** esophageal carcinoma, gastroesophageal junction adenocarcinoma, PD-1 inhibitor, efficacy, safety

## Abstract

Esophageal cancer (EC) is a lethal disease, and ranks 7th in incidence and 6th in mortality worldwide. Patients are treated with surgery and/or chemoradiotherapy for a curative intent, but for those with advanced diseases systemic chemotherapy and targeted therapy are the mainstay treatment with poor prognosis. For the patients with squamous cell carcinoma and those progressed after chemotherapy, treatment option is even fewer, and effective treatment modalities are urgently needed. Preclinical and clinical studies have found the PD-1/PD-L1 inhibitors activate T lymphocytes, inhibit cancer growth, and improve survival in cancer patients. Multiple PD-1/PD-L1 inhibitors have been approved for the management of a variety of cancers. Interestingly, a large of proportion of EC patients have tumors with PD-L1 expression and high tumor mutation burden. Trials have been performed to evaluate the efficacy and safety of the PD-1/PD-L1 inhibitors in EC patients. This review will summarize the current progress in this field, especially the toxicities associated with these agents.

## Introduction

Esophageal cancer (EC) is a dismal disease, with an estimated 5-year survival rate of only 20%. Histologically, this disease entity is categorized to squamous cell carcinoma (SCC) and adenocarcinoma. In 2018, 572,034 new cases and 508,585 deaths were reported worldwide (Bray et al., 2018). Either surgery alone or with peri-operative chemotherapy is a curative treatment modality for locally advanced stage. For those in their late stages, systemic chemotherapy and targeted therapy are the mainstay treatment (Abdo et al., 2017). Platinum-based chemotherapy regimens, commonly combined with fluoropyrimidine or taxane, are the main treatment, with disappointing objective response rate (ORR) of 23.2% to 60.6%, high incidence of adverse event, and a short overall survival (OS) of 7.7 to 15.5 months. And for the SCC patients and those progressed during or after chemotherapy, the treatment options are more limited. Single-agent chemotherapy, such as paclitaxel, docetaxel, and irinotecan, was recommended, resulting in an ORR of 20% and poor OS of approximately 5 months (Shi et al., 2013; Wang et al., 2013; Shirakawa et al, 2014; Prithviraj et al., 2015; Liu et al., 2016; Hiramoto et al., 2018). In summary, the existing treatments for EC have a limited efficacy and severe adverse events. Effective treatment modalities with moderate adverse event are urgently needed (Thallinger et al., 2011).

Lines of direct and indirect evidence show that the interaction between PD-1 and PD-L1 inhibits the function of T lymphocytes to evade persistent inflammatory or autoimmune reaction. However, this protective mechanism is hijacked by the tumors to escape the immune surveillance through upregulating PD-L1 expression on tumor cells (Mcdermott and Atkins, 2013; Araki et al., 2014; Guillebon et al., 2015; Chen and Han, 2015). Preclinical and clinical studies have found the PD-1/PD-L1 inhibitors activate T lymphocytes. And activated T lymphocytes help to inhibit cancer growth, and improve survival in cancer patients. PD-1/PD-L1 inhibitors have been approved for the management of a variety cancers, such as melanoma, lung cancer, and renal cell cancer etc. (Weber et al., 2015; Chedgy and Black, 2016; Reck et al., 2016). The efficiency of the PD-1/PD-L1 inhibitors is related to the PD-L1 expression, and/or tumor mutation burden (TMB) in tumor cells (Topalian et al., 2012; Rosenberg et al., 2016; Yarchoan et al., 2017; Hellmann et al., 2018a; Hellmann et al., 2018b; Hellmann et al., 2018c; Rizvi et al., 2018; Keenan et al., 2019). Interestingly, a large proportion of EC patients have tumors with PD-L1 expression (14.5–82.8%, in different reports) and high TMB (Lawrence et al., 2013; Hsieh et al., 2018). Not surprisingly, trials have been initiated to evaluate the efficacy and safety of the PD-1/PD-L1 inhibitors in EC patients.

To this end, four antibodies (pembrolizumab, nivolumab, toripalimab, and camrelizumab) were tested in EC patients. Pembrolizumab and nivolumab are authorized globally for a dozen of cancers, including non-small cell lung cancer, head and neck squamous cell carcinoma, urothelial carcinoma, and so on. Toripalimab and camrelizumab are available in China with the indication for melanoma and classical Hodgkin lymphoma, respectively. Pembrolizumab and nivolumab had similar pharmacokinetic parameters. But no published data on that of toripalimab and camrelizumab are available now. Up to now, no clinical trial to directly compare these antibodies regarding safety and tolerability was reported. One report inferred pembrolizumab and nivolumab had similar safety profile (Wang et al., 2019). Data on direct comparison of clinical efficacy for these antibodies are lacking. This review provided a brief summary of current progress of these antibodies in the field of EC treatment, especially the toxicities associated with these agents.

### Data Acquisition

The electronic database including PubMed, Clinical trials (https://clinicaltrials.gov/), Embase, Web of science, Cochrane library were retrieved by using the Keywords “esophageal cancer,” “esophageal carcinoma,” “immunotherapy,” “PD-1,” “PD-L1,” “clinical trial.” The literature in abstract form was viewed, and those with only protocol design or preliminary results were excluded. Finally, 12 studies involving PD-1 inhibitor monotherapy with full description of the outcome were selected.

### Pembrolizumab

Pembrolizumab is a humanized IgG4 antibody for PD-1. In a pilot phase 1b study, KEYNOTE-012, pembrolizumab was first tested in patients with PD-L1-positive recurrent or metastatic gastroesophageal junction (GEJ) and gastric adenocarcinoma, without limitation on the number of lines of previous therapy (Muro et al., 2016). Thirty-nine patients were enrolled and received pembrolizumab 10 mg/kg every 2 weeks. The ORR was 22%. Median progression-free survival (mPFS), median OS (mOS), and duration of response (DOR) were 1.9 months (mo), 11.4 mo, and 40 weeks. Treatment-related adverse events (TRAEs) occurred in 67% patients, grade 3 or 4 TRAEs in 13% patients (n = 5). Fatigue (18%), decreased appetite (13%), hypothyroidism (13%), pruritus (13%), and arthralgia (10%) were the most common TRAEs. Grade 3–4 TRAEs included grade 3 fatigue (n = 2), grade 3 pemphigoid (n = 1), grade 3 hypothyroidism (n = 1), grade 3 peripheral sensory neuropathy (n = 1), and grade 4 pneumonitis (n = 1). There were no treatment-related death or discontinuation of drugs due to TRAE.

Phase 2 trial KEYNOTE-059 investigated the efficacy and safety of pembrolizumab monotherapy in the late (≥3) lines of therapy (Fuchs et al., 2018). Two hundred fifty-nine patients with similar features as those in KEYNOTE-012 study were enrolled, except for no requirement of PD-L1 expression. Pembrolizumab was given every 3 weeks (at fixed dose of 200 mg). The ORR for the intention-to-treatment (ITT) cohort was 11.6%, and in PD-L1-positive and -negative cohorts, it was 15.5% and 6.4%, respectively. The mDOR for ITT, PD-L1-positive, and PD-L1-negative patients was 8.4 mo, 16.3 mo, and 6.9 mo, respectively. The mPFS and mOS of ITT patients were 2 mo and 5.6 mo. TRAEs of any grade and grade 3–5 occurred in 60.2% and 17.8% patients. Fatigue, pruritus, rash, hypothyroidism, decreased appetite, anemia, nausea, diarrhea, and arthralgia were the most common TRAEs. There were two treatment-related deaths and two cases of treatment-related discontinuation.

The efficacy of pembrolizumab in the second-line therapy was tested in a randomized controlled phase 3 trial KEYNOTE-061 (Shitara et al., 2018). Five hundred ninety-two patients with advanced GEJ or gastric adenocarcinoma who progressed after chemotherapy regimen of fluoropyrimidine and platinum were enrolled. Pembrolizumab (200 mg) every 3 weeks for up to 2 years or paclitaxel 80 mg/m^2^ on days 1, 8, 15 in a 4-week cycle was administered. In population with PD-L1 ≥ 1% (PD-L1 CPS ≥ 1), the mOS of pembrolizumab and chemotherapy was 9.1 and 1.5 mo. And mPFS was 8.3 and 4.1 mo, respectively. The ORR of pembrolizumab and chemotherapy was 16% and 14%, and mDOR was 18 and 5.2 mo. In the ITT population, TRAEs occurred in 53% and 84% patients receiving pembrolizumab and chemotherapy, and for grade 3–5 TRAEs, the incidence was 14% and 35%. The most common grade 3–5 TRAE for pembrolizumab were anemia and fatigue. Three percent of the patients in pembrolizumab group discontinued treatment because of TRAEs. The mortality rate was 1% in pembrolizumab group.

KEYNOTE-062 was a phase 3 trial to investigate pembrolizumab with (p+c) or without (p) chemotherapy versus chemotherapy (c, cisplatin, and fluoropyrimidine) for the first-line treatment (Tabernero et al., 2019). This study was also conducted in the GEJ and gastric adenocarcinoma with PD-L1 CPS ≥ 1. Totally, 763 patients were enrolled. Pembrolizumab monotherapy compared with chemotherapy did not show any survival benefit. In patients with PD-L1 CPS ≥ 10, pembrolizumab monotherapy showed improved mOS over chemotherapy (17.4 and 10.8 mo), but inferior mPFS (2.9 and 6.1 mo) and ORR (25% and 36.7%). P+c vs c did not show any benefit in OS and PFS regardless of patients PD-L1 CPS status (CPS ≥ 1 or CPS ≥ 10). Grade 3–5 TRAE rates were 17% (p), 73% (p+c), and 65% (c).

Pembrolizumab was also tested in other histological types, mainly SCC. KEYNOTE-028 was a phase 1b study similar to KEYNOTE-012 study, to explore the efficacy and safety of pembrolizumab in late-line treatment (87% patients had received ≥2 lines of treatment) for all histological types (including SCC, adenocarcinoma, (Doi et al., 2018). Twenty-three patients with PD-L1-positive tumors were enrolled. The incidence rate of TRAEs was 39%, and the mOS was 7 months.

A phase 2 trial KEYNOTE-180 was similar to KEYNOTE-059 study, investigating pembrolizumab monotherapy in the setting of late (≥3) lines of therapy (Shah et al., 2019). But this study recruited patients of all histological types. PD-L1(+) was mandatory, defined as CPS ≥ 10. One hundred twenty-one patients were enrolled. The ORR was 14.3% among SCC patients, and 5.2% among adenocarcinoma patients. The mPFS and mOS were 2 and 5.8 mo. Subgroup analysis showed mOS was better in patients with SCC. The incidence rates of TRAEs and grade 3–5 TRAEs were 57.9% and 12.4%, respectively. The most commonly TRAEs included fatigue, rash, pruritus, hypothyroidism, and diarrhea. Treatment-related discontinuation (n = 5) and death (n = 1) were reported.

KEYNOTE-181 was a phase 3 trial similar to KEYNOTE-061, where pembrolizumab was used in the second line of therapy, except for recruitment of all histotypes (Kojima et al., 2019). Six hundred twenty-eight patients were enrolled. In the ITT population, pembrolizumab compared with chemotherapy did not show significant benefit in mOS and mPFS. But in subgroup of patients with PD-L1 CPS ≥ 10, pembrolizumab treatment led to longer mOS over chemotherapy (9.3 and 6.7 mo) with statistical significance. The ORR was also improved (21.5% and 6.1%) in this subpopulation. The incidence rate of TRAEs of pembrolizumab and chemotherapy were 64.3% and 86.1%. The incidence rates of grade 3–5 TRAEs were 18.2% and 40.7%, respectively. There was no significant difference in treatment-related discontinuation (6.1% vs 6.4%) and death (1.5% vs 1.7%).

### Nivolumab

Nivolumab is another humanized IgG4 monoclonal antibody for PD-1 immune checkpoint. For patients with advanced GEJ and gastric adenocarcinoma, nivolumab was tested in trial ATTRACTION-2 (in Asia) and CheckMate-032 (in Western countries).

ATTRACTION-2 was a randomized, double-blind, placebo-controlled, phase 3 trial to evaluate the efficacy and safety of nivolumab in heavily pretreated adenocarcinoma (Kang et al., 2017). Four hundred ninety-three patients were enrolled and were randomly assigned (2:1) to receive nivolumab 3 mg/kg or placebo every 2 weeks. The mOS of nivolumab and placebo was 5.26 and 4.14 mo, and the mPFS was 1.61 and 1.45 mo. The ORR and mDOR of nivolumab were 11.2% and 9.53 mo. The incidence of TRAEs and grade 3–5 TRAEs of nivolumab was 43% and 10%. The common TRAEs included pruritus, diarrhea, rash, and fatigue. In the nivolumab group, nine cases of treatment discontinuation and five deaths occurred.

Conducted in a cohort with similar demographic features, CheckMate-032 was a phase 1/2 trial where nivolumab monotherapy or nivolumab plus ipilimumab were administered (Janjigian et al., 2018). One hundred sixty patients were enrolled. The treatment consisted three arms: either nivolumab 3 mg/kg every 2 weeks (n = 59), or nivolumab 1 mg/kg plus ipilimumab 3 mg/kg every 3 weeks for four cycles (N1I3, n = 49), or nivolumab 3 mg/kg plus ipilimumab 1 mg/kg every 3 weeks for four cycles (N3I1, n = 52). After four cycles, all patients were maintained on nivolumab 3 mg/kg therapy. For the group of nivolumab monotherapy, N1I3, and N3I1, the ORR was 12%, 24% and 8%. The mOS was 6.2, 6.9, 4.8 mo, and the mPFS was 1.4, 1.4, and 1.6 mo. The incidence rates of TRAEs and grade 3–4 TRAEs of these groups were 69%, 17%, 84%, and 47%, 75%, 27%, respectively. The most frequently occurred TRAEs were fatigue, pruritus, rash, diarrhea, decreased appetite, and increased transaminase. The incidence rates of discontinuation of drug related to TRAEs were 3%, 20%, and 13% in three groups.

Another phase 2 study ATTRACTION-1 was conducted in Japan, where patients with EC were enrolled (Kudo et al., 2017). Sixty-five patients with heavily treated SCC were enrolled and received nivolumab 3 mg/kg every 2 weeks. The ORR was 17%, mOS was 10.8 months, and mPFS was 1.5 months. The incidence rates of TRAEs and grade 3 or worse TRAEs were 60% and 17%, respectively. The most common adverse events were diarrhea, decreased appetite, constipation, rash, and fatigue. Seven patients discontinued therapy due to TRAEs, and no death related to TRAE occurred.

### Toripalimab (JS001) and Camrelizumab (SHR1210)

Toripalimab and camrelizumab are two of Chinese domestic me-too antibodies in this class. A phase 1b/2 trial (Clinicaltrial identifier: NCT02915432) evaluated the efficacy and safety of toripalimab in refractory/metastatic esophageal SCC (Xu et al., 2018). Fifty-six patients were enrolled and received toripalimab at the dose of 3 mg/kg every 2 weeks. Till September 2017, 34 patients were evaluated, and 8 patients achieved partial response with an ORR of 23.5%. TRAEs were mostly grade 1 or 2. Another trial (NCT02742935) was a dose-escalating phase 1 study investigating the efficacy and safety of camrelizumab in ≥2 line treatment of esophageal SCC (Huang et al., 2018). The dose was given at 60, 200, and 400 mg every 2 weeks. The ORR was 33.3% and the mPFS was 3.6 months. The incidences of TRAEs and grade 3 TRAEs were 83.3% and 10%, respectively. The most common TRAEs included reactive capillary hemangiomas, pruritus, hypothyroidism, and fever. There was no treatment-related discontinuation due to toxicity.

## Discussion

**Figure 1:**
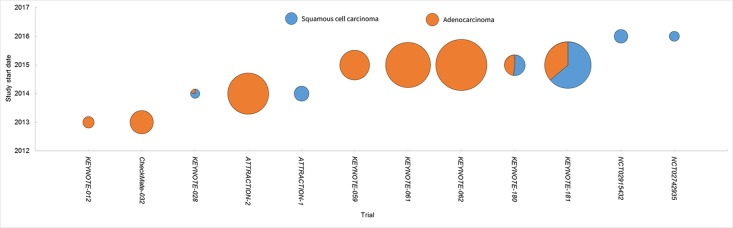
Clinical studies of PD-1 inhibitors in EC. Each trial was plotted against the year of the initiation. The circle area denoted the sample size, and SCC and adenocarcinoma were depicted in different colors.

EC is a lethal disease affecting millions of people worldwide. Histologically, it is composed of two main subtypes, i.e., SCC and adenocarcinoma. They differ to a large extent in their genetic aberrations, epidemiology, etiology, and clinical manifestations. Thus, the two subtypes should have distinct strategy of therapy. Previously radio- and chemo-therapy remain the mainstay of the therapy for those unsuitable for surgery. Targeted therapy including anti-angiogenesis agents and epidermal growth factor receptor inhibitors obtains authorization for the treatment of adenocarcinoma, but not SCC. Therefore, there is a large unmet need for the improvement of SCC treatment. It is in high expectance that the immune checkpoint inhibitors help to advancing the progress in this field. Our summary showed that most of the studies were performed in adenocarcinoma till now, but the trends toward SCC became obvious (**Figure 1**).

**Figure 2:**
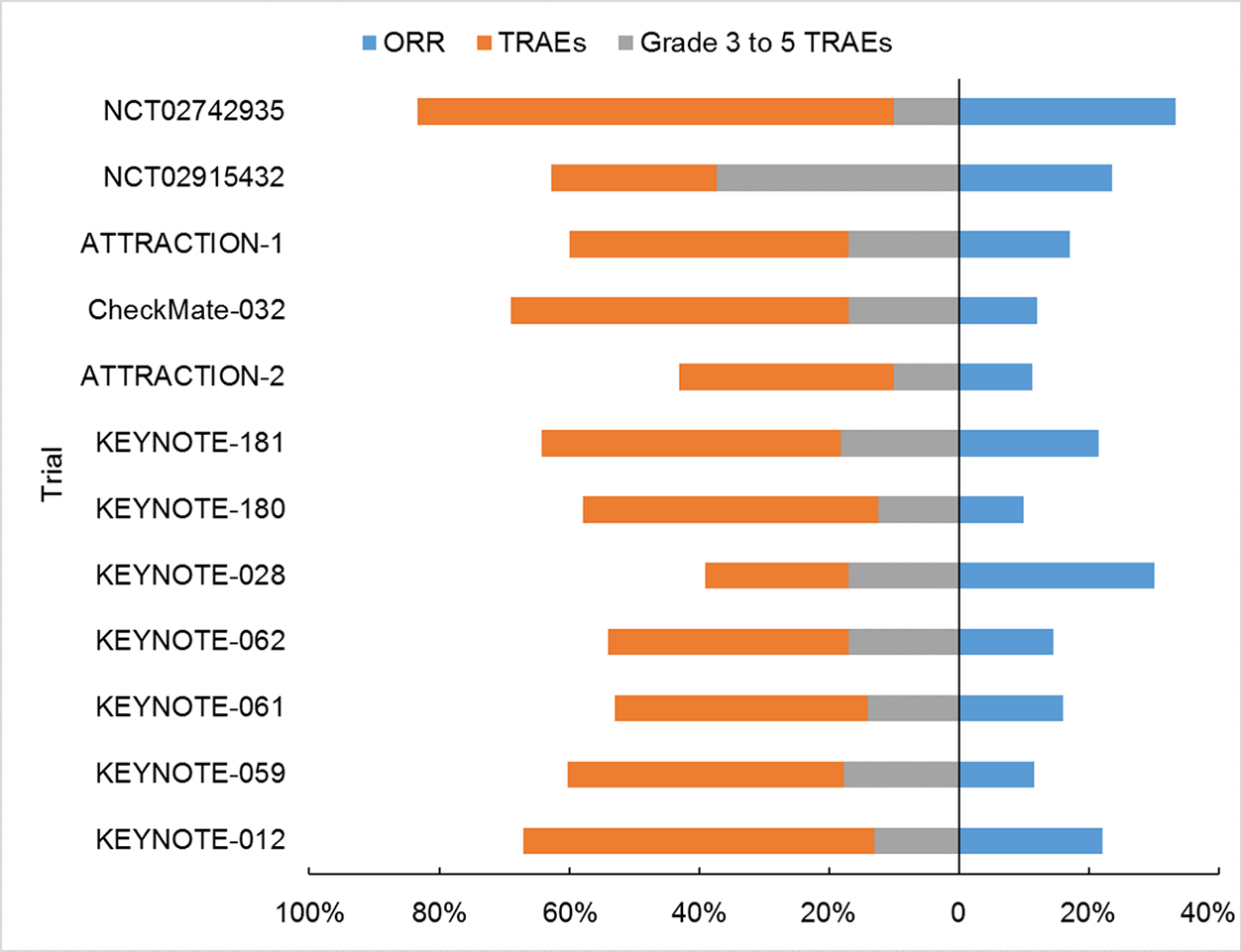
Summary of toxicities and ORRs in each study.

The current review summarized 12 trials on PD-1 inhibitor monotherapy for the treatment of advanced EC, including phase 3 (n = 4) and phase 1/2 trials (n = 8). Among them, KEYNOTE-062 is the only one investigating pembrolizumab monotherapy in the first-line treatment. Both KEYNOTE-061 and KEYNOTE-181 investigated the efficacy of pembrolizumab monotherapy in the second-line treatment. The rest nine trials investigated efficacy and safety of PD-1 inhibitors in late lines. The immune checkpoint inhibitors showed promising results, with minimal to mild toxicities (**Figure 2**). TRAEs in EC were similar to those reported in other solid tumors, and no unexpected TRAEs occurred (Topalian et al., 2012; Garon et al., 2015; Ferris et al., 2016; Tomita et al., 2017).

It was interesting to compare the PD-1/PD-L1 inhibitors and traditional chemotherapy for EC patients. The two modalities were compared in a head-to-head fashion in three of the trials (**Table 1**). Although relative a small sample, a clear trend could be easily found favoring the former, with elevated ORRs, prolonged PFS and OS, and less frequency of any-grade or grade 3–5 AE in immunotherapy.

**Table 1:** The comparison of PD-1 inhibitors and chemotherapy.

Trial	Drug	PD-L1 CPS	ORR (%)	DOR (mo)	PFS (mo)	OS (mo)	AEs (%)	AE ≥Grade 3 (%)
		<1	2 vs 10.4	NA	NA	4.8 vs 8.2		
KEYNOTE-061	Pembro vs Chemo	≥1	16 vs 14	8 vs 5.2	1.5 vs 4.1	9.1 vs 8.3	53 vs 84	14 vs 35
		≥10	24.5 vs 9.1	NA	NA	10.4 vs 8		
KEYNOTE-062	Pembro vs Chemo	≥1	14.5 vs 36.8	NA	2 vs 6.4	10.6 vs 11.1	54.3 vs 91.8	17 vs 69
		≥10	25 vs 36.7	NA	2.9 vs 6.1	17.4 vs 10.8		
KEYNOTE-181	Pembro vs Chemo	All comer	13.1 vs 6.7	8.5 vs 10.7	2.1 vs 3.4	7.1 vs 7.1	64.3 vs 86.1	18.2 vs 40.9
		≥10	21.5 vs 6.1	9.3 vs 7.7	2.6 vs 3	9.3 vs 6.7		

Pembrolizumab, nivolumab, and toripalimab had similar incidence of TRAE (**Figure 2**, 39%–67%, 43%–60%, 62.7%, respectively), lower than that of camrelizumab (83.3%). Also, grade 3–5 TRAEs seemed less likely in pembrolizumab (12.4%–18.2%), nivolumab (10%–17%), camrelizumab (10%) than toripalimab (37.3%). The incidence of immune-related adverse events (irAEs) was 18% to 26% in pembrolizumab, 10.2% in toripalimab, and 83.3% in camrelizumab. It should be noted the toxicities of toripalimab and camrelizumab were both extracted from small-sized, phase 1 studies, and might be over-estimated.

Next, specific AE was analyzed. Because the information was lacking for toripalimab and camrelizumab, only pembrolizumab and nivolumab were compared. A consistent higher incidence was observed in hyperthyroidism (3.5%–7.7% and 1%), hypothyroidism (7.4%–12.8% and 0), pneumonitis (1.9%–4.9% and 0.3%), colitis (1%–2.6% and 1%), and hepatitis (0.4%–2.6% and 0) for pembrolizumab than nivolumab. For the severe (grade 3–5) irAE, pembrolizumab also had worse record in hypothyroidism (0.4%–2.5% and 0), pneumonitis (0.3%–2.6% and 0.3%), colitis (0.3%–1.2% and 0.3%), and hepatitis (0.4%–1% and 0) than nivolumab. But it was imprudent to make direct comparison of data from different trials. For EC treatment, these four agents had comparable safety and efficiency, based on the direct comparison of their reported outcomes (**Figure 2**). This conclusion also got supports from the biochemical features of these drugs. They are monoclonal antibodies blocking PD-1, and they have the same, if any difference, of action mechanism.

From these trials, one reasons that PD-1 inhibitors would play a role in the treatment of advanced EC. But the question is when and how to apply these agents appropriately. At this time point, Food and Drug Administration (FDA) authorized pembrolizumab for the late (≥2) line treatment for the cancer patients whose tumors harbor high TMB, irrespective of tissue origin, also including those with EC. Additionally, FDA approved pembrolizumab for the 2-line treatment for the patients with SCC with CPS ≥ 10 and for the 3-line treatment for the patients of with GEJ and gastric adenocarcinoma with PD-L1 CPS ≥ 1. Based on the encouraging results, PD-1 inhibitor combined with chemotherapy for the first-line therapy for EC is in underway (**Table 2**).

**Table 2:** Ongoing phase 2/3 trial with PD-1 inhibitor combined with chemotherapy in first-line treatment of esophageal carcinoma.

Trial	Phase	Status	Drug	Tumor	Treatment
KEYNOTE-590—China Extension Study	3	Recruiting	Pembrolizumab+Cisplatin+5-FU/Placebo+Cisplatin+5-FU	Esophageal Carcinoma	First-line
KEYNOTE-590	3	Active	Pembrolizumab+Cisplatin+5-FU/Placebo+Cisplatin+5-FU	Esophageal Carcinoma	First-line
NCT02954536	2	Recruiting	Pembrolizumab +Trastuzumab+ Chemotherapy (Capecitabine/5-Fluorouracil+Cisplatin/Oxaliplatin)	Esophagogastric Carcinoma	First-line
NCT03342937	2	Recruiting	Pembrolizumab + Oxaliplatin +Capecitabine	Esophagogastric Carcinoma	First-line
NCT03615326	3	Recruiting	Pembrolizumab+Trastuzumab+Chemotherapy/Placebo+Trastuzumab+Chemotherapy (Capecitabine/5-Fluorouracil/S-1+Cisplatin/Oxaliplatin)	Gastroesophageal junction and gastric adenocarcinoma	First-line
Checkmate 648	3	Recruiting	Nivolumab + Ipilimumab/Nivolumab + Cisplatin + Fluorouracil/Cisplatin + Fluorouracil	Esophageal Carcinoma	First-line
NCT03409848	3	Recruiting	Nivolumab and Trastuzumab +Ipilimumab/FOLFOX	Esophagogastric Carcinoma	First-line
NCT03829969	3	Recruiting	JS001 +paclitaxel +cisplatin/placebo +paclitaxel +cisplatin	Esophageal Squamous Cell Carcinoma	First-line
NCT03691090	3	Recruiting	SHR-1210 + paclitaxel + cisplatin/placebo +paclitaxel +cisplatin	Esophageal squamous cell carcinoma	First-line
NCT03603756	2	Recruiting	SHR-1210 + Apatinib+ Chemotherapy (irinotecan/paclitaxel+ nedaplatin)	Esophageal Squamous Cell Carcinoma	First-line

## Conclusion

In general, PD-L1 inhibitor monotherapy in the treatment of pretreated EC has a promising antitumor activity and manageable toxicity.

## Author Contributions

Z-YD and YH contributed conception and overall idea of the study. YH wrote the first draft of the manuscript. Z-YD wrote sections of the manuscript. Both authors contributed to manuscript revision, read and approved the submitted version.

## Conflict of Interest

The authors declare that the research was conducted in the absence of any commercial or financial relationships that could be construed as a potential conflict of interest.
